# High-Nitrate-Supply-Induced Transcriptional Upregulation of Ascorbic Acid Biosynthetic and Recycling Pathways in Cucumber

**DOI:** 10.3390/plants12061292

**Published:** 2023-03-13

**Authors:** Neda Hesari, Anita Szegő, Iman Mirmazloum, Zsolt Pónya, Erzsébet Kiss-Bába, Henriett Kolozs, Márta Gyöngyik, Dominika Vasas, István Papp

**Affiliations:** 1 Department of Plant Physiology and Plant Ecology, Institute of Agronomy, Hungarian University of Agriculture and Life Sciences, Ménesi Str. 44, 1118 Budapest, Hungary; 2 Division of Applied Food Crop Production, Department of Agronomy, Institute of Agronomy, Hungarian University of Agricultural and Life Sciences, Guba Sándor Str. 40, 7400 Kaposvár, Hungary; 3 Agricultural and Food Research Centre, Széchenyi István University, Egyetem tér 1, 9026 Győr, Hungary

**Keywords:** antioxidants, ascorbate, *Cucumis sativus*, oxidative stress, nitrate

## Abstract

Nowadays open field and protected vegetable cultivation practices require and use genotypes which are precisely tailored to their intended growth environments. Variability of this kind provides a rich source of material to uncover molecular mechanisms supporting the necessarily divergent physiological traits. In this study, typical field-optimized and glasshouse-cultivated cucumber F1 hybrids were investigated, and displayed slower growth (‘Joker’) and faster growth (‘Oitol’) in seedlings. Antioxidant capacity was lower in ‘Joker’ and higher in ‘Oitol’, pointing to a potential redox regulation of growth. The growth response of seedlings to paraquat treatment indicated stronger oxidative stress tolerance in the fast-growing ‘Oitol’. To test whether protection against nitrate-induced oxidative stress was also different, fertigation with increasing potassium nitrate content was applied. This treatment did not change growth but decreased the antioxidant capacities of both hybrids. Bioluminescence emission revealed stronger lipid peroxidation triggered by high nitrate fertigation in the leaves of ‘Joker’ seedlings. To explore the background of the more effective antioxidant protection of ‘Oitol’, levels of ascorbic acid (AsA), as well as transcriptional regulation of relevant genes of the Smirnoff–Wheeler biosynthetic pathway and ascorbate recycling, were investigated. Genes related to AsA biosynthesis were strongly upregulated at an elevated nitrate supply in ‘Oitol’ leaves only, but this was only reflected in a small increase in total AsA content. High nitrate provision also triggered expression of ascorbate–glutathion cycle genes with stronger or exclusive induction in ‘Oitol’. AsA/dehydro–ascorbate ratios were higher in ‘Oitol’ for all treatments, with a more pronounced difference at high nitrate levels. Despite strong transcriptional upregulation of ascorbate peroxidase genes (*APX*) in ‘Oitol’, APX activity only increased significantly in ‘Joker’. This suggests potential inhibition of APX enzyme activity specifically in ‘Oitol’ at a high nitrate supply. Our results uncover an unexpected variability in redox stress management in cucumbers, including nitrate inducibility of AsA biosynthetic and recycling pathways in certain genotypes. Possible connections between AsA biosynthesis, recycling and nitro-oxidative stress protection are discussed. Cucumber hybrids emerge as an excellent model system for studying the regulation of AsA metabolism and the roles of AsA in growth and stress tolerance.

## 1. Introduction

The cucumber (*Cucumis sativus*) is an important vegetable worldwide, with an estimated total production over 90 million metric tons in 2020. In commercial cultivation, a large variety of F1 hybrids is grown [1]. Two major groups of genotypes are field and greenhouse-optimized hybrids. In both groups intensity of growth is a significant trait. Fast-growing genotypes, often intended for a soilless medium in a protected environment, should tolerate concentrated nutrients in a small-volume root system. Due to this and other differently adapted traits, the available genotypes may serve as a rich resource for physiological and molecular studies.

Ascorbic acid (AsA) has fundamental functions in shaping the growth and redox balance of plants, with a well-documented role in stress tolerance [2,3] as a potent water-soluble antioxidant, contributing to reactive oxygen species (ROS) scavenging under oxidative stress. In addition to maintaining redox homeostasis, AsA may also have an impact through stress-related hormonal balance as well. AsA is known to be synthesized in plants in several ways, of which the Smirnoff–Wheeler pathway is the most dominant [4]. External application of AsA-promoted growth in several cases which have been studied, while overexpression of the relevant biosynthetic genes could also increase growth and improve stress tolerance [5]. Redox-related effects of AsA can be complex. In addition to protecting membrane lipids [6], AsA may be specifically involved in reactions affecting photosynthesis, regulation of cell cycle, influencing cell wall metabolism or its physical properties and apoplastic pH or gibberellic acid synthesis [4,7,8,9,10]. The connection between ascorbic acid and growth promotion may primarily lie in redox regulation, but links between cell wall metabolism and ascorbate biosynthesis may also play a role.

Nowadays, intensive fertigation is a typical horticultural practice. Nitrogen as a major nutrient is commonly delivered in the form of nitrate. An excessive nitrate supply may inhibit growth if it exceeds genotype specific thresholds. This effect has been well documented, often leading to growth retardation in shoot and root [11,12,13]. In addition to a range of metabolic perturbations [14], nitro-oxidative effects were implicated as an underlying mechanism in high nitrate stress [15]. An extended supply of nitrate fertigation triggers a complex redox response including reactive nitrogen and oxygen species which interact through various ways and at multiple levels [16,17]. Fast-growing hybrids bred for covered cultivation are generally subject to a continuous and high-level nutrient supply; therefore it is hypothesized that they tolerate nutrient stresses better. The main purpose of this work is to find differences between representative hybrids of the two major cultivation types in processes related to oxidative stress management in high nitrate fertigation conditions.

Here we present the results of comparative experiments with two contrasting commercial F1 cucumber hybrids. The growth of the hybrids as well as their tolerance against oxidative and nitro-oxidative stresses were characterized. Young plants were exposed to paraquat and to elevated levels of nitrate fertigation. Growth parameters and some indicators of nitrate assimilation and oxidative stress were recorded and compared. As a major antioxidant, ascorbic acid biosynthesis was investigated at the transcriptional and metabolite levels. To this end, the expression of some genes implicated to participate in the main AsA biosynthesis pathway and in AsA recycling was studied under increasing concentrations of nitrate fertigation.

## 2. Results

### 2.1. Physiological and Biochemical Measurements

**Figure 1:**
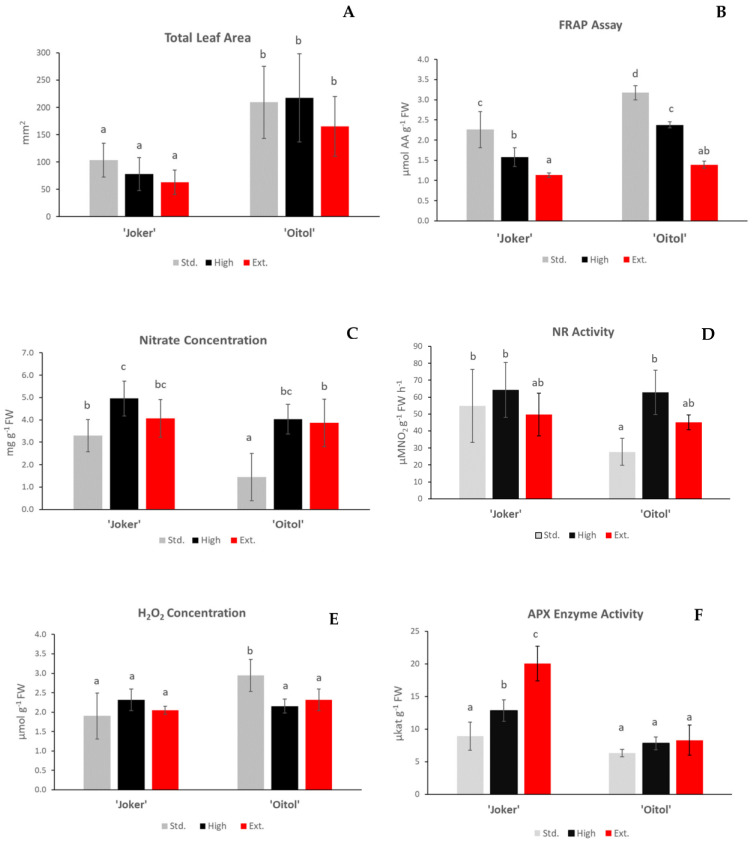
Total leaf area (**A**), Antioxidant capacity (**B**), Nitrate content (**C**), Nitrate reductase activity (**D**), Hydrogen peroxide content (**E**) and APX enzyme activity (**F**) in the leaves of two cucumber hybrid lines treated with different nitrate concentrations. Std.: 4 mM, High: 20 mM and Ext.: 50 mM KNO_3_. Different letters are for significantly different values in accordance with the ANOVA followed by Tukey’s post hoc test (*p* < 0.05). Data are the means of at least six replicates, with error bars representing standard deviations.

Leaf surface area has been found to be a sensitive indicator of growth in cucumbers. Following this parameter, ’Oitol’ grew faster than ’Joker’, and growth was not accelerated by increasing the KNO_3_ level of the fertigation solution in either case (Figure 1A). The antioxidant capacity (AOC) of the leaf tissues was evaluated by means of FRAP values comparison. The AOC of ’Oitol’ plants was significantly higher at standard and high nitrate levels than that of ‘Joker’ plants (Figure 1B). In both hybrid lines AOC decreased linearly in response to increased nitrate supplementation. In accordance with expectations, the concentration of NO_3_^-^ in leaves was increased in High (20 mM) compared to Standard level (4 mM) nitrate treatments in both hybrids (Figure 1C). Standard-treated ’Oitol’ consistently gave the lowest nitrate concentration values of all samples. There were no statistically significant differences among nitrate reductase activities in leaves, except for Standard-nitrate-treated ’Oitol’ which gave consistently low values (Figure 1D). No major differences could be observed for H_2_O_2_ levels (Figure 1E) except, that the values of Standard ’Oitol’ were consequently higher than those of the other samples.

The total ascorbate peroxidase (APX) activity was only significantly affected (*p* < 0.05) and increased in ‘Joker’ in response to elevated nitrate concentration, whereas ‘Oitol’ leaves showed no significant changes in their APX activity (Figure 1F).

**Figure 2:**
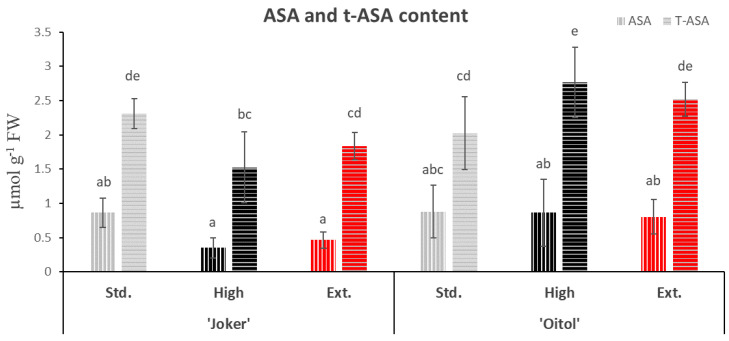
Tissue concentrations of reduced (AsA) and total ascorbic acid (t-AsA) in the leaves of two F1 cucumber hybrid lines treated with different nitrate concentrations. Std.: 4 mM, High: 20 mM and Ext.: 50 mM KNO_3_. Different letters are for significant differences (*p* < 0.05). Data are the means of at least six replicates, with error bars representing standard deviations.

The total ascorbate content of ‘Joker’ leaves decreased in plants treated with High nitrate (Figure 2). However, the total ascorbic acid content of leaves was significantly higher (*p* < 0.05) in ‘Oitol’ plants treated with High (20 mM) KNO_3_ when compared with Standard (4 mM) treatment (Figure 2). The ratios of total AsA to dehydroascorbic acid (DHA) were found to be higher in ‘Oitol’ than in ‘Joker’ for all treatments (Joker: Std, 0.6; High, 0.3; Ext, 0.3 and Oitol: Std, 0.75; High, 0.44; Ext, 0.46). Differences were more pronounced at High and Extreme nitrate treatments (1.47× and 1.44× increased ratios, respectively) compared to Standard (1.27× increased ratio).

Paraquat (PQ) treatment at Standard nitrate provision resulted in a stronger reduction of growth in ‘Joker’ than in ‘Oitol’. This was confirmed by some growth parameters measured, such as root length, root fresh weight and length of hypocotyl (Table 1). PQ inhibited root growth by 86.3% in ‘Joker’, but only by 77.5% in ‘Oitol’ plants in accordance with their total length. The same trend was observed for the root fresh weight with a 73% and 65% reduction in ‘Joker’ and ‘Oitol’, respectively. Turning to hypocotyl length, when treated with 10 µM methyl viologen ‘Joker’ suffered a 21% reduction while ‘Oitol’ only showed a 2% shorter length.

**Figure 3:**
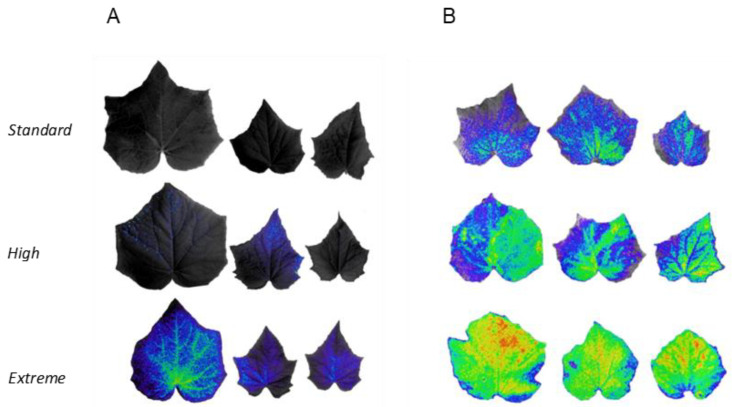
Typical pseudo-colored images based on bioluminescence of typical (**A**) ‘Oitol’ and (**B**) ‘Joker’ cucumber leaves treated with different levels of nitrate concentrations. The photocount-comparison is depicted by pixel peak distributions on 2 D-images represented by pseudo color-coded pixel intensity.

Ultra-weak bio photon emission analysis-based high-resolution images revealed a significantly different emission intensity between the two hybrid lines studied. Bio photon emission increased in response to an elevated nitrate supply in both hybrids. Emission was strikingly higher at all treatments in ‘Joker’, implicating that the higher oxidative burden was reflected in the images, pointing to the fact that the ‘Joker’ hybrid may contain more lipid peroxidation products under elevated nitrate exposure (Figure 3), while the ‘Oitol’ hybrid may have fewer lipid peroxidation products under elevated nitrate exposure. The time course of overall photon count measurements showed the same pattern and tendency, with the lowest values being for the ‘Oitol’ treated with 4 mM KNO_3_ and the highest values being for ‘Joker’ treated with 50 mM KNO_3_ throughout the period investigated (Figure S1).

### 2.2. RT-PCR Analysis of Smirnoff–Wheeler AsA biosynthetic Pathway and Ascorbate–Glutathion Cycle Genes

**Figure 4:**
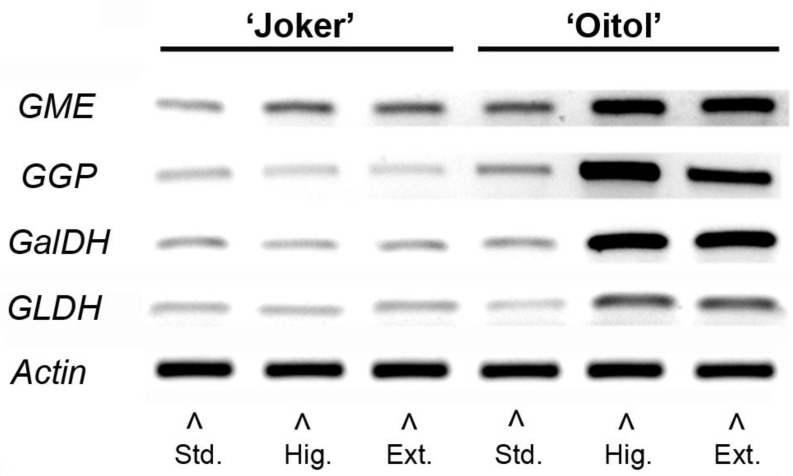
Semiquantitative RT-PCR expression analysis of Smirnoff–Wheeler AsA biosynthetic pathway genes in the leaves of two F1 cucumber hybrid lines treated with different nitrate concentrations. *GME*: GDP-mannose-3′,5′-epimerase; *GGP*: GDP-galactose phosphorylase; *GalDH*: l-galactose dehydrogenase; *GLDH*: galactono-1,4-lactone dehydrogenase.

The expression of important and well-known genes involved in the Smirnoff–Wheeler AsA biosynthetic pathway was investigated for their transcript levels in leaf samples of ‘Joker’ and ‘Oitol’ plants treated with different nitrate concentrations. In accordance with semi-quantitative RT-PCR, all studied genes: GDP-mannose-3′,5′-epimerase (*GME*), GDP-galactose phosphorylase (*GGP*), l-galactose dehydrogenase (*GalDH*) and galactono-1,4-lactone dehydrogenase (*GLDH*), were upregulated exclusively or predominantly in the ‘Oitol’ hybrid at high and extreme nitrate fertigation (Figure 4).

**Figure 5:**
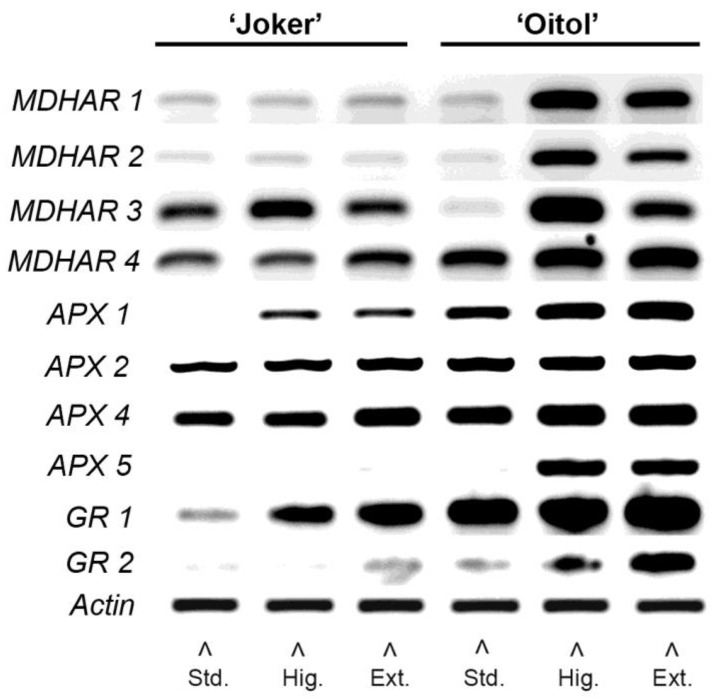
Semiquantitative RT-PCR expression analysis of ascorbate–glutathion cycle genes in the leaves of two F1 cucumber hybrid lines treated with different nitrate concentrations. *MDHAR*: Monodehydroascorbate reductase; *APX*: Ascorbate peroxidase; *GR*: Glutathione reductase.

RT-PCR analysis also approved the transcriptional induction of representative genes of the ascorbate–glutathion cycle: monodehydroascorbate reductases (*MDHAR*), ascorbate peroxidases (*APX*) and glutathione reductases (*GR*) in both hybrids (Figure 5). The expression levels were typically higher in ‘Oitol’ than in ‘Joker’. The expression of only two *MDHAR* genes was induced in ‘Joker’ while all four *MDHAR* genes investigated were upregulated in ‘Oitol’ at higher nitrate fertigation. The four *APX* genes showed significantly increased expression at elevated nitrate levels, and this was more pronounced in ‘Oitol’. The *GR* expressions were also upregulated in response to an elevated nitrate supply, with their highest expression being detected in ‘Oitol’ plants treated with the highest concentration of nitrate (50 mM).

## 3. Discussion

Representative hybrids of two basic cultivation types of cucumber were studied. ‘Joker’ is a slower-growing, field-optimized genotype, while ‘Oitol’ is intended for covered cultivation and exhibits fast growth (Figure 1A, Standard treatment).

Redox regulation is a well-established concept of growth control [10,18]. In our study, significantly higher (*p* < 0.05) reducing capacity was found in the leaves of fast-growing ‘Oitol’ than in ‘Joker’ (Figure 1B, Standard treatment). To assess whether increased reducing capacity coincides with protection against oxidative stress, paraquat (PQ) tolerance was measured. Indeed, improved PQ tolerance was found in ‘Oitol’ when compared to ‘Joker’ (Table 1). This confirms that higher antioxidant capacity as well as oxidative stress tolerance coincide with faster growth in ‘Oitol’, lending further support for a potential role of redox regulation in development.

Nowadays, as part of commercial cultivation practices, cucumber plants are often fertilized intensively. The root systems of greenhouse-grown hybrids are normally confined to a small volume of space, where they are subjected to high nutrient concentrations. This necessitates a high tolerance level in greenhouse-adapted hybrids against overdoses of fertilizers, e.g., nitrate stress. High-salinity fertigation [12] and excessive nitrate supply in particular cause inhibition of growth [13,14], with several underlying mechanisms being implicated, including oxidative stress [19,20] which is also observed in cucumbers [16]. To test whether the oxidative stress tolerance of ‘Oitol’ is coupled with nitro-oxidative stress tolerance, fertigation with increasing potassium nitrate content was applied to the genotypes in this study.

Fertigation solution was applied at three nitrate concentrations: Standard (4 mM), High (20 mM) and Extreme (50 mM). Total leaf area measurements approved the faster growth of ‘Oitol’ with all treatments (Figure 1A). The growth rate was not affected significantly by any nitrate levels applied for this hybrid. Leaf nitrate concentrations were established at the three leaves developmental stage of seedlings (Figure 1C). At Standard supply, the tissue concentration of nitrate was higher in the field-optimized ‘Joker’ than in ‘Oitol’. This indicates a more efficient uptake/transport system from the roots, which may be a useful adaptive trait when nitrate is taken up from more diluted solutions in the soil. At this supply, ‘Oitol’ reached only moderate nitrate and nitrate reductase levels in shoot, however growth of the plant was more intensive at the same time. A potential explanation may be a higher rate of nitrate assimilation in the roots of ‘Oitol’, and this possibility deserves further investigation. The partitioning of nitrate assimilation between root and shoot is known as a major trait in the environmental adaptation of plants [21]). At high nitrate supply (50 mM), nitrate levels in leaf tissues reached a plateau. This may be explained by the tight control of nitrate uptake, potentially occurring through feedback repression of transport [22]. Nitrate reductase (NR) activity mirrored the nitrate concentration, reflecting their well-established regulatory connection [23] (Figure 1D).

Hydrogen peroxide concentration remained relatively constant through treatments, except for standard-nitrate-supplied ‘Oitol’ (Figure 1E). At this fertigation level, higher H_2_O_2_ concentration coincided with lower NR activity in ‘Oitol’, indicating the potential involvement of NR in regulating antioxidant mechanisms. This effect has been implicated to occur through nitric oxide (NO) formation, which in turn is thought to potentiate antioxidant responses [24,25]. H_2_O_2_ levels did not increase even at extreme nitrate levels, indicating that plants can maintain H_2_O_2_ equilibrium under high nitrate stress. This effect had already been demonstrated by Bellegarde and co-workers [26], who found similar H_2_O_2_ levels in low- vs. high-nitrate-treated wild-type *Arabidopsis*. This equilibrium is probably due to complex interactions between reactive oxygen and nitrogen species effects/signaling. Unlike H_2_O_2_, other ROS (lipid peroxides) were specifically affected by the nitrate stress applied (see below).

Antioxidant capacity was estimated by the FRAP method, with ‘Oitol’ displaying significantly higher values than the corresponding ‘Joker’ plants for all treatments. Both hybrids showed a decreasing trend of free reducing capacity towards higher nitrate supply levels (Figure 1B). This indicates that increasing amounts of antioxidants are being used up to balance the oxidative effects stemming from nitrate supplementation.

The bioluminescence method was applied to assess signs of oxidative burden in leaves,. Bioluminescence is attributed to resolution of excited states in oxidative reactions, thought to be specifically indicative of lipids peroxidation [26]. Increasing nitrate provision triggered biophoton emission with characteristic differences between the two hybrids. Bioluminescence was emitted from Standard to Extreme nitrate supply in ‘Joker’ with increasing intensity (Figure 3). However, the signal was only detected at considerably higher nitrate levels in ‘Oitol’, approving its increased redox protection.

Reductants of different kinds may contribute to the superior reducing capacity of ‘Oitol’, as demonstrated in this study. It has been proposed that high ascorbic acid content may be responsible for eliminating lipid peroxides [27], as sources of the bioluminescence signal. The mitigating effect of AsA on lipid peroxidation has also been documented by Ali and co-workers [28]. Therefore, in the following, focus was put on AsA as a reductant which also has a potential link to growth promotion. In earlier works, growth rate has been associated with AsA, based on external applications leading to accelerated growth [29]. In transgenic studies, overexpression of AsA biosynthetic enzymes and related regulators provided evidence for the growth-promoting and stress-ameliorative effects of AsA [30]. Therefore, AsA levels were measured in leaf tissues of both hybrids. Total AsA concentrations were only significantly different between the hybrids at High nitrate supply, favoring ‘Oitol’. AsA levels only increased from Standard to High treatment in ‘Oitol’ (Figure 2). The concentrations measured were in the same range as was reported by Wu et al. [31] for cucumber seedlings. The ratio of AsA to dehydro-ascorbic acid was higher in ‘Oitol’ than in ‘Joker’ at all treatments.

Transcription of genes coding for key enzymes of the major AsA biosynthetic pathway and recycling [32] were also investigated. Expression of genes for GDP-d-mannose epimerase (*GME*), GDP-l-galactose phosphorylase (*GGP*), l-galactose dehydrogenase (*GalDH*) and l-galactono-1,4-lactone dehydrogenase (*GLDH*) were induced by high and extreme levels of nitrate fertigation with striking intensity in ‘Oitol’ (Figure 4). This indicates a strong and coordinated transcriptional upregulation of the Smirnoff–Wheeler (SW) pathway in response to nitrate treatment in a genotype-dependent manner. Transcriptional induction of genes for ascorbate biosynthesis is not a commonly considered effect of nitrate provision according to meta-analyses based on Arabidopsis data [33]. Hybrid specificity of this upregulation may lead to new clues to understanding regulatory aspects of ascorbate biosynthesis. High-level transcriptional induction in ‘Oitol’ is in sharp contrast with the modest total AsA concentration increase in leaf tissues from Standard to High nitrate levels. Several scenarios may explain this discrepancy. It can be hypothesized that feedback regulation, potentially at GGP translation [5], may limit ascorbate levels in ‘Oitol’. In another, not-mutually-exclusive scenario, divergent turnover rates of synthesis and degradation may occur in the two hybrids, resulting in similar steady-state levels of total AsA. Faster replenishment of the AsA pool may allow its increased availability for antioxidant reactions, explaining the mitigation of lipid peroxidation in ‘Oitol’.

To shed further light on AsA recycling and its regulation, expression of key genes of the ascorbate glutathione cycle was investigated. Transcription of genes coding for monodehydroascorbate reductase (MDHAR), ascorbate peroxidase (APX) and glutathione reductase (GR) enzymes were strongly induced by nitrate treatments at higher level or exclusively in ‘Oitol’ (Figure 5). APX enzyme activity was also studied. Data surprisingly revealed that, despite strong transcriptional upregulation of *APX* genes in ‘Oitol’, APX activity was only increased in ‘Joker’ (Figure 5). MDHAR upregulation indicated increased AsA regeneration, which together with decreased APX activity may have resulted in a higher AsA/DHA ratio, which was indeed found in ‘Oitol’ compared to ‘Joker’. APX enzymes are known subjects of regulation by nitration and nitrosylation [34,35]. The direct impact of this effect on AsA/DHA balance, however, is difficult to fully reconcile as other enzymes of the ascorbate–glutathione cycle may also be the subject of similar (or antagonistic) posttranslational regulations by reactive nitrogen species [36]. Nevertheless, an effect of nitrate on AsA metabolism through nitric oxide (NO) has been confirmed by earlier reports [19]. Thus, it is hypothesized that downregulation of APX activity at high nitrate levels may be mediated by NO, which may occur on a larger scale in ‘Oitol’. The precise mechanism of this effect needs to be clarified by further studies.

## 4. Materials and Methods

### 4.1. Plant Material and Growth Conditions

The open-field-grown cucumber F1 cultivar hybrid ‘Joker’ and the greenhouse-grown ‘Oitol’ were considered and used in this research. Seeds of ‘Joker’ and ‘Oitol’ were obtained from ZKI Ltd., Kecskemét, Hungary and Semillas Fito Co., Barcelona, Spain, respectively. To establish cultures, cucumber seeds were submerged in 100 mL of distilled water for 24 h at 25 °C to imbibe.

Plants were grown in a semi-hydroponic growth system, which approximates commercial cultivation practice in soilless media. For each treatment, three seeds were planted in 7.5 × 7.5 × 6.5 cm rockwool cubes (in quadruplicate) which were inserted in 20-cm-diameter pots containing 120 g of perlite. Pots were transferred to a growth chamber (FitoClima 600, Rio de Mouro, Portugal) where the seeds germinated and grew at 26 ± 1 °C and 20 ± 1 °C, day and night temperatures respectively, under 16 h photoperiod with a photosynthetic photon flux density (PPFD) of 150 μmol·m^−2^·s^−1^ at culture level (provided by cool-white fluorescent lamps) and at 75–80% of relative humidity. Fertigation treatments were applied every other day after the cotyledons expanded and the first leaf emerged. Parts of the plants’ root systems grew in the rockwool cubes and extensively penetrated the surrounding perlite, which stood in a pool of fertigation solution.

The treatments were continued for 21 days, after which leaves from each individual plant in all treatments systems were harvested and photographed, and the total leaf area per plant was calculated with the ImageJ software (version 1.53t, Image Processing and Analysis in Java, Bethesda, MD, USA). The data obtained were then statistically analyzed. Fully expanded leaves were deep frozen in liquid nitrogen and stored at −80 °C for further molecular analysis. All subsequent experiments were performed at least three times on different biological materials, unless otherwise indicated.

### 4.2. Fertigation Treatments

Nitrate was supplied at three different concentrations (Standard 4 mM, High 20 mM and Extreme 50 mM) in the form of KNO_3_ in a fertigation solution (prepared in deionized water) assembled for cucumbers [37]. For a full description of treatment solutions, see Table S1. Pots were flashed with 250 mL of fresh fertigation solution every other day. On the fertigation day intervals, the pH of the treatment solution pools under the pots was measured and adjusted to 5.8–6.0 with 1 M citric acid.

### 4.3. Determination of Nitrate Content in Leaves

The nitrate content of leaves was determined with the salicylic–sulphuric acid method in accordance with the ISO standard [38]. One g of cucumber leaves was ground and immersed in a small flask containing 40 mL deionized water. The mixture was heated on a boiling water bath for 15 min and 2 mL of Carrez solution I (15% K_4_(Fe(CN)_6_) × 3H_2_O) and 2 mL of Carrez solution II (22% Zn acetate) were added to precipitate interfering colloids and proteins. The flask was filled up to 100 mL with deionized water. After filtering, filtrate was used for the spectrophotometric determination. For each 5 mL sample, 1 mL of 0.5% Na-salicylate was added and the fluid was evaporated to dryness in a 100 °C oven. The residue was allowed to cool at room temperature, and dissolved in 1 mL of sulfuric acid, then in 7 mL of 10 M NaOH solution. The flask was filled up to 50 mL with deionized water. The yellow complex formed by nitration of salicylic acid under strong acidic conditions was photometered at 410 nm. The nitrate content was determined using a calibration line prepared with a standard solution of sodium nitrate.

### 4.4. Nitrate Reductase Activity

The activity of nitrate reductase enzyme was estimated in the 9th hour of daylight by the method used by Kim and Seo [39]. Plant leaf samples (200 mg) were homogenized in 750 μL extraction buffer containing 250 mM Tris-HCl (pH 8.0), 1 mM ethylenediaminetetraacetic acid, 1 µM Na_2_MoO_4_, 5 µM FAD, 3 mM dithiothreitol, 1% BSA, 12 mM 2-mercaptoethanol and 250 µM phenylmethylsulfonyl fluoride. The homogenate was centrifuged (20 min, 13,000 rpm, 4 °C) and the supernatant was decanted. The reaction mixture contained 150 μL of crude enzyme extract and 850 μL reaction buffer (40 mM NaNO_3_, 80 mM Na_2_HPO_4_, 20 mM NaH_2_PO_4_, 0.2 mM NADH). The reaction was carried out at room temperature for 2 h. Subsequently, 200 μL of 1% sulphanilamide and 200 μL of 0.05% N-(1-naphthyl) ethylene diamine hydrochloride were added to the mixture and incubated at room temperature for 15 min. Enzyme activity was measured at 540 nm in a spectrophotometer and expressed in µM NO_2_ g^−1^ FW h^−1^.

### 4.5. H_2_O_2_ Levels

The H_2_O_2_ content of the leaves was measured at 560 nm in a spectrophotometer, based on colorimetric reaction as previously described by Kellős et al. [40]. Briefly, 200 mg of plant leaf samples were homogenized in 1 mL 10% phosphoric acid. The supernatant was used for determination of H_2_O_2_. The sample extract (50 μL) was mixed with 950 μL of reaction mixture containing 100 µM Xylenol Orange, 250 µM ammonium ferrous sulphate, 100 µM sorbitol and 25 µM sulfuric acid for H_2_O_2_ analysis. H_2_O_2_ was used for calibration.

### 4.6. Antioxidant Capacity (FRAP Assay)

The level of antioxidant capacity in the leaf tissues of cucumber plants was evaluated according to Oszlányi et al. [41]. Two-hundred mg of leaves was homogenized in 2 mL of 70% ethanol. The homogenate was centrifuged at 13,000 rpm for 10 min at 4 °C. The reaction mixture contained 50 μL of supernatant and 250 μL of FRAP reagent (10 mM TPTZ solution in 40 mM HCl; 20 mM FeCl_3_ and 0.3 M acetate buffer, pH 3.6 in 1:1:10 ratio). The reaction was monitored for 10 min in a spectrophotometer at 593 nm. The FRAP value of the samples was given based on a calibration curve in µM ascorbate equivalent ev. g^−1^ FW.

### 4.7. Determination of Ascorbic Acid Content

The ascorbate content of the leaves was measured according to Tari et al. [42]. Total ascorbate (T-AsA) was determined after reduction of dehydroascorbate (DHA) to ascorbate (AsA) with dithiothreitol (DTT), and the concentration of DHA was estimated from the difference between T-AsA and AsA. Two-hundred mg of leaves was homogenized with 0.8 mL of 5% trichloroacetic acid (TCA). The homogenate was centrifuged at 13,000 rpm for 20 min at 4 °C and the 50 μL supernatant was used for further investigation. To assay total ascorbate, 50 μL of 10 mM (DTT) and, after 10 min of incubation at room temperature, 50 μL of 0.5% N-ethylmaleimid (NEM) were added. To assay ascorbate, 100 μL of distilled water was added instead of DTT and NEM. Then, 250 μL of 10% TCA was added to both samples. To determine ascorbate, the extract was mixed with 200 μL of 43% H_3_PO_4_, 200 μL of 4% 2-bipyridyl, 200 μL 3% FeCl_3_ and incubated for 60 min at 37 °C. Ascorbate concentrations were determined spectrophotometrically at 525 nm. Calculations were made based on a standard curve in the range of 0–250 µg AsA ml^−1^.

### 4.8. Determination of Ascorbate Peroxidase Activity

Ascorbate peroxidase (APX; EC: 1.11.1.1) activity was assayed in accordance with de Azevedo Neto et al. [43] with minor modifications by monitoring the rate of ascorbate oxidation at 290 nm. Samples (0.2 g) of cucumber leaves were extracted with 0.6 mL of 100 mM potassium phosphate buffer pH 7.0 containing 0.1 mM EDTA and centrifuged at 13,000 rpm for 15 min at 4 °C. The reaction mixture (1.0 mL) contained 50 mM phosphate buffer (pH 6.0), 0.1 µM EDTA, 0.5 mM ascorbate and 1.0 mM H_2_O_2_. Reaction was triggered by adding 10 μL of extract supernatant and the changes in absorbance were read at 290 nm after 3 min of incubation at room temperature. The enzyme activity was expressed using the molar extinction coefficient of ascorbate (2.8 mM^−1^·cm^−1^).

### 4.9. Paraquat Dichloride Treatment

Seeds of the two different hybrids (‘Oitol’ and ‘Joker’) were treated with (10 µM) methyl viologen (Paraquat dichloride) during early seedling growth. In brief, the seeds were submerged in deionized water overnight and were placed on wet filter papers in glass Petri dishes (18 Ø cm) in a growth chamber at an average temperature of 26 °C and a light intensity of 150 μmol·m^−2^·s^−1^ until the emergence of radicles. The Paraquat treatment was started by adding/refreshing 20 mL of either deionized water (control) or methyl viologen (PQ; treatment) at two day intervals under the same conditions. Morphological parameters (hypocotyl length, root length and root FW) were recorded and the results are presented as means ± standard deviations (*n* ≤ 6).

### 4.10. Ultra-Weak Bio Photon Emission

Leaves of cucumbers grown under different nitrate supply levels were subjected to UPE (ultra-weak photon emission) imaging. Intact leaves of approximately the same size were separated from the plants and placed in the NightShade LB 985 Plant Imaging System (Berthold Technologies, Bad Wildbad, Germany). Luminescence emission of photons from the plants were visualized using a thermoelectrically cooled (−70 °C) CCD camera (NightOWLcam, Berthold Technologies) mounted on a dark, light-proof chamber. A back-lit, midband-coated full-frame chip with a spectral range of 350–1050 nm (quantum efficiency: 90% at 620 nm) was employed for photon detection and XY-imaging. To increase detection sensitivity, the variable binning was set to 2 × 2 resulting in final resolutions of 512 × 512 pixels and 26 × 26 µm² pixel size (slow scan mode). The exposure time was set to 60 s and the images were analyzed with the IndiGo software (Version 2.0.5.0, Berthold Technologies, Bad Wildbad, Germany). The presented images are a representative selection from the series of photos with the highest detected signal intensity level for each treatment. The experiment was repeated twice on different biological samples.

### 4.11. RNA Isolation, cDNA Synthesis, RT-PCR

Deep-frozen leaf samples in liquid N_2_ (0.5 g each) were homogenized in sterile mortar and pestles for total RNA extraction following a CTAB-based protocol [44]. The RNA integrity was confirmed on an EcoSafe-stained agarose gel (1%). RNA was quantified by NanoDrop 1000 spectrophotometer at 260 nm. All samples were treated with DNase I (Thermo Scientific, Waltham, MA, USA) to eliminate genomic DNAs, then RNA concentrations were normalized to 5 μg/30 μL for all reaction mix. DNase-treated total RNA quality was confirmed on 1% agarose gel prior to reverse transcription. First-strand cDNAs were synthesized by RT-PCR using 5 μg of total RNA as template and M-MuLV RT enzyme using a Maxima Reverse Transcriptase kit (Thermo Scientific) with oligo (dT)_20_ primers in accordance with the given protocol. Primers of cucumber ascorbate peroxidases and relevant genes of the Smirnoff–Wheeler pathway along with a control actin gene (Table S2) were tested for PCR amplification with GO Taq G2 DNA polymerase (Promega, Madison, WI, USA). The selected *Actin* gene and its stable expression in similar experiments had been confirmed earlier in our other contributions [41,45] and remained steady in the normalized RNA and cDNA samples in this work. Amplification was achieved in a Master Cycler instrument (Eppendorf AG, Hamburg, Germany), applying 3 min at 95 °C and 28 cycles of 15 s: 95 °C, 30 s: 56–60 °C, 30 s: 72 °C and a final extension for 7 min at 72 °C. Amplified fragments from genomic DNA and cDNAs were visualized on 1.2% (*w*/*v*) ethidium bromide-stained agarose gel in 1 × TBE.

### 4.12. Statistical Methods

The recorded data were analyzed using SPSS (IBM SPSS Statistics Version 27.0 IBM Corp, Armonk, NY, USA) software and the results are shown as mean values with standard deviations among at least two biological and technical replicates. Normality of the residuals was confirmed by Shapiro–Wilk’s test. The homogeneity of variances was checked by Levene’s test. The differences among the means were evaluated by one-way ANOVA followed by Tukey’s post hoc HSD test and considered significant at (*p* < 0.05. MS Excel 2017 was used for the charts data representations.

## 5. Conclusions

The presented results demonstrate the different redox properties of two cucumber hybrids with contrasting growth habits. Fast-growing ‘Oitol’ is equipped with high antioxidant capacity, which probably helped to effectively mitigate paraquat-induced oxidative stress. High-nitrate-supply-induced oxidative stress manifested in lipid peroxidation in leaves. This occurred at a lower level in ‘Oitol’ compared to the ‘Joker’ hybrid that possessed lower antioxidant capacity. At high nitrate provision, robust induction of AsA biosynthesis and recycling-related genes was observed, predominantly in ‘Oitol’. Despite transcriptional upregulation of *APX* genes, APX enzyme activity remained uninduced in ‘Oitol’. This hybrid displayed elevated AsA/DHA ratios, which could result from lower APX activity. Although multiple genes of the AsA biosynthetic pathway were strongly induced in ‘Oitol’, only a moderate increase in the total AsA level was found. We put forward two potential explanations for this: it can be hypothesized that in ‘Oitol’ a known translational-level regulation mechanism limits AsA biosynthetic capacity (see above for details). It may also be feasible that the observed steady-state levels do not reflect the actual turnover rates of AsA that might be substantially different between the hybrids. Nevertheless, our results potentially offer a method of achieving transcriptional activation of the AsA biosynthetic pathway using an easilyavailable inducer. This possibility has far-reaching biotechnological implications. Moreover, cucumber hybrids clearly emerge as an appropriate model system for studying regulation of AsA biosynthesis as well as the roles of AsA in growth and stress tolerance.

## Figures and Tables

**Table 1:** Effect of Paraquat dichloride on seedling characteristics of two cucumber hybrids.

Treatments	Hypocotyl Length (cm)	Cotyledon FW (g)	Root Length (cm)	Root FW (g)
‘Joker’ Control	1.4 ± 0.17 b	0.18 ± 0.03 a	76.4 ± 11.09 b	0.23 ± 0.02 c
‘Joker’ PQ	1.1 ± 0.1 a	0.18 ± 0.04 a	10.4 ± 5.87 a	0.06 ± 0.01 a
‘Oitol’ Control	1.98 ± 0.16 c	0.20 ± 0.02 a	97.0 ± 7.15 c	0.13 ± 0.04 b
‘Oitol’ PQ	1.94 ± 0.13 c	0.20 ± 0.01 a	21.8 ± 6.14 a	0.04 ± 0.01 a

Different letters in each column are for significantly different values in accordance with the ANOVA followed by Tukey’s post hoc test (*p* < 0.05). Data are the means of at least six replicates, ±standard deviations. FW: fresh weight.

## Data Availability

The data presented in this study are available on request from the corresponding author.

## Supplementary Materials

The following supporting information can be downloaded at: https://www.mdpi.com/article/10.3390/plants12061292/s1, Figure S1: Time course of overall photon count per second (CPS) values measured on ‘Oitol’ and ‘Joker’ leaves treated with different levels of nitrate concentrations; Table S1: Composition of the fertigation solutions used for treatments; Table S2: Target genes, oligonucleotide primers and expected product sizes in RT-PCRs [46].

## References

[1] Welbaum G.E. (2015). Vegetable Production and Practices.

[2] Gallie D.R. (2013). L-ascorbic acid: A multifunctional molecule supporting plant growth and development. Scientifica.

[3] Rosado-Souza L., Fernie A.R., Aarabi F. (2020). Ascorbate and Thiamin: Metabolic Modulators in Plant Acclimation Responses. Plants.

[4] Smirnoff N. (2018). Ascorbic acid metabolism and functions: A comparison of plants and mammals. Free Radic. Biol. Med..

[5] Paciolla C., Fortunato S., Dipierro N., Paradiso A., De Leonardis S., Mastropasqua L., de Pinto M.C. (2019). Vitamin C in Plants: From Functions to Biofortification. Antioxidants.

[6] Shalata A., Neumann P.M. (2001). Exogenous ascorbic acid increases resistance to salt stress and reduces lipid peroxidation. J. Exp. Bot..

[7] Ivanov B.N. (2014). Role of ascorbic acid in photosynthesis. Biochemistry.

[8] Smirnoff N. (2000). Ascorbic acid: Metabolism and functions of a multi-facetted molecule. Curr. Opin. Plant Biol..

[9] Fenech M., Amaya I., Valpuesta V., Botella M.A. (2019). Vitamin C content in fruits: Biosynthesis and regulation. Front. Plant Sci..

[10] Pignocchi C., Foyer C.H. (2003). Apoplastic ascorbate metabolism and its role in the regulation of cell signalling. Curr. Opin. Plant Biol..

[11] Kappel N., Boros I.F., Ravelombola F.S., Sipos L. (2021). EC sensitivity of hydroponicallygrown lettuce (*Lactuca sativa* L.) types in terms of nitrate accumulation. Agriculture.

[12] Chen B.M., Wang Z.H., Li S.X., Wang G.X., Song H.X., Wang X.N. (2004). Effects of nitrate supply on plant growth, nitrate accumulation, metabolic nitrate concentration and nitrate reductase activity in three leafy vegetables. Plant Sci..

[13] Tian Q., Chen F., Liu J., Zhang F., Mi G. (2008). Inhibition of maize root growth by high nitrate supply is correlated with reduced IAA levels in roots. J. Plant Physiol..

[14] Saiz-Fernández I., De Diego N., Brzobohatý B., Muñoz-Rueda A., Lacuesta M. (2017). The imbalance between C and N metabolism during high nitrate supply inhibits photosynthesis and overall growth in maize (*Zea mays* L.). Plant Physiol. Biochem..

[15] Xu H., Sun X., Shi Q., Yang F., Yang X., Wang X. (2012). Physiological responses of two cucumber cultivars to nitrate stress. J. Plant Nutr..

[16] Chaki M., Begara-Morales J.C., Barroso J.B. (2020). Oxidative stress in plants. Antioxidants.

[17] Begara-Morales J.C., Sánchez-Calvo B., Chaki M., Valderrama R., Mata-Pérez C., Padilla M.N., Corpas F.J., Barroso J.B. (2016). Antioxidant systems are regulated by nitric oxide-mediated post-translational modifications (NO-PTMs). Front. Plant Sci..

[18] Kocsy G., Tari I., Vanková R., Zechmann B., Gulyás Z., Poór P., Galiba G. (2013). Redox control of plant growth and development. Plant Sci..

[19] Vidal A., Cantabella D., Bernal-Vicente A., Díaz-Vivancos P., Hernández J.A. (2018). Nitrate-and nitric oxide-induced plant growth in pea seedlings is linked to antioxidative metabolism and the ABA/GA balance. J. Plant Physiol..

[20] Bellegarde F., Maghiaoui A., Boucherez J., Krouk G., Lejay L., Bach L., Gojon A., Martin A. (2019). The Chromatin Factor HNI9 and ELONGATED HYPOCOTYL5 Maintain ROS Homeostasis under High Nitrogen Provision. Plant Physiol..

[21] Andrews M. (1986). The partitioning of nitrate assimilation between root and shoot of higher plants. Plant Cell Environ..

[22] Li J.Z., Li B., Guan Q., Gao J.M. (2022). Signal molecules controlling nitrate uptake by roots. J. Plant Interact..

[23] Campbell W.H. (1999). Nitrate reductase structure, function and regulation: Bridging the gap between biochemistry and physiology. Annu. Rev. Plant Physiol. Plant Mol. Biol..

[24] Correa-Aragunde N., Foresi N., Lamattina L. (2015). Nitric oxide is a ubiquitous signal for maintaining redox balance in plant cells: Regulation of ascorbate peroxidase as a case study. J. Exp. Bot..

[25] León J., Costa-Broseta Á. (2020). Present knowledge and controversies, deficiencies, and misconceptions on nitric oxide synthesis, sensing, and signaling in plants. Plant Cell Environ..

[26] Birtic S., Ksas B., Genty B., Mueller M.J., Triantaphylidès C., Havaux M. (2011). Using spontaneous photon emission to image lipid oxidation patterns in plant tissues. Plant J..

[27] Szarka A., Tomasskovics B., Bánhegyi G. (2012). The ascorbate-glutathione-α-tocopherol triad in abiotic stress response. Int. J. Mol. Sci..

[28] Ali B., Pantha S., Acharya R., Ueda Y., Wu L.B., Ashrafuzzaman M., Ishizaki T., Wissuwa M., Bulley S., Frei M. (2019). Enhanced ascorbate level improves multi-stress tolerance in a widely grown indica rice variety without compromising its agronomic characteristics. J. Plant Physiol..

[29] Akram N.A., Shafiq F., Ashraf M. (2017). Ascorbic acid-a potential oxidant scavenger and its role in plant development and abiotic stress tolerance. Front. Plant Sci..

[30] Chaturvedi S., Khan S., Bhunia R.K., Kaur K., Tiwari S. (2022). Metabolic engineering in food crops to enhance ascorbic acid production: Crop biofortification perspectives for human health. Physiol. Mol. Biol. Plants.

[31] Wu Y., Hu L., Liao W., Dawuda M.M., Lyu J., Xie J., Feng Z., Calderón-Urrea A., Yu J. (2019). Foliar application of 5-aminolevulinic acid (ALA) alleviates NaCl stress in cucumber (*Cucumis sativus* L.) seedlings through the enhancement of ascorbate-glutathione cycle. Sci. Hortic..

[32] Bulley S., Laing W. (2016). The regulation of ascorbate biosynthesis. Curr. Opin. Plant Biol..

[33] Canales J., Moyano T.C., Villarroel E., Gutiérrez R.A. (2014). Systems analysis of transcriptome data provides new hypotheses about Arabidopsis root response to nitrate treatments. Front. Plant Sci..

[34] Begara-Morales J.C., Sánchez-Calvo B., Chaki M., Valderrama R., Mata-Pérez C., López-Jaramillo J., Padilla M.N., Carreras A., Corpas F.J., Barroso J.B. (2014). Dual regulation of cytosolic ascorbate peroxidase (APX) by tyrosine nitration and S-nitrosylation. J. Exp. Bot..

[35] González-Gordo S., Rodríguez-Ruiz M., López-Jaramillo J., Muñoz-Vargas M.A., Palma J.M., Corpas F.J. (2022). Nitric Oxide (NO) Differentially Modulates the Ascorbate Peroxidase (APX) Isozymes of Sweet Pepper (*Capsicum annuum* L.) Fruits. Antioxidants.

[36] Begara-Morales J.C., Sánchez-Calvo B., Chaki M., Valderrama R., Mata-Pérez C., Padilla M.N., Corpas F.J., Barroso J.B. (2015). Modulation of the ascorbate–glutathione cycle antioxidant capacity by posttranslational modifications mediated by nitric oxide in abiotic stress situations. Reactive Oxygen Species and Oxidative Damage in Plants Under Stress.

[37] Nutritional Recommendations for Cucumber. https://www.haifa-group.com/crop-guide/vegetables/cucumber.

[38] International Organization for Standardization (1984). ISO 6635, 1984: Fruits, Vegetables and Derived Products—Determination of Nitrite and Nitrate Content.

[39] Kim J.Y., Seo H.S. (2018). In vitro nitrate reductase activity assay from Arabidopsis crude extracts. Bio-protocol.

[40] Kellős T., Tímár I., Szilágyi V., Szalai G., Galiba G., Kocsy G. (2008). Stress hormones and abiotic stresses have different effects on antioxidants in maize lines with different sensitivity. Plant Biol..

[41] Oszlányi R., Mirmazloum I., Pónya Z., Szegő A., Jamal S., Bat-Erdene O., Papp I. (2020). Oxidative stress level and dehydrin gene expression pattern differentiate two contrasting cucumber F1 hybrids under high fertigation treatment. Int. J. Biol. Macromol..

[42] Tari I., Csiszár J., Horváth E., Poór P., Takács Z., Szepesi A. (2015). The alleviation of the adverse effects of salt stress in the tomato plant by salicylic acid shows a time- and organ-specific antioxidant response. Acta Biol. Cracov. Bot..

[43] De Azevedo Neto A.D., Prisco J.T., Enéas-Filho J., Abreu C.E.B.D., Gomes-Filho E. (2006). Effect of salt stress on antioxidative enzymes and lipid peroxidation in leaves and roots of salt-tolerant and salt-sensitive maize genotypes. Environ. Exp. Bot..

[44] Jaakola L., Pirttilä A.M., Halonen M., Hohtola A. (2001). Isolation of high quality RNA from bilberry (*Vaccinium myrtillus* L.) fruit. Appl. Biochem. Biotechnol.-Part B Mol. Biotechnol..

[45] Szegő A., Mirmazloum I., Pónya Z., Bat-Erdene O., Omran M., Kiss-Bába E., Gyöngyik M., Papp I. (2021). Downregulation of polyamine and diamine oxidases in silicon-treated cucumber. Plants.

[46] Liu P., Li Q., Gao Y., Wang H., Chai L., Yu H., Jiang W. (2019). A New perspective on the effect of UV-B on L-ascorbic acid metabolism in cucumber seedlings. J. Agric. Food Chem..

