# Supplementary material for: High-Nitrate-Supply-Induced Transcriptional Upregulation of Ascorbic Acid Biosynthetic and Recycling Pathways in Cucumber
Source: Plants (Basel). 2023 Mar 13;12(6):1292. doi: 10.3390/plants12061292 (PMC10051573; doi:10.3390/plants12061292)
Supplement: Supplementary file 1 [file plants-12-01292-s001.zip › plants-2186694-supplementary.pdf]

Supplementary data.

High nitrate supply induced transcriptional upregulation of ascorbic acid biosynthetic and recycling pathways in cucumber

Neda Hesari, Anita Szegő, Iman Mirmazloum, Zsolt Pónya, Erzsébet Kiss-Bába, Henriett Kolozs, Márta Gyöngyik, Dominika Vasas, István Papp

Table S1. Different concentrations of potassium nitrate in fertigation solutions

	**Standard**	**High**	**Extreme**
**KNO_3**	**4 mM**	**20 mM**	**50 mM**
$MgSO_4$		<0.5 mM>	
NaFe-EDTA		<5 µM>	
KH_2PO_4		<1 mM>	
H_3BO_3		<0.2 µM>	
Na_2MoO_4		<10.0 µM>	
$ZnSO_4$		<1.0 µM>	
$MnCl_2$		<2.0 µM>	
$CuSO_4$		<0.5 µM>	
$CoCl_2$		<0.2 µM>	
$NiSO_4$		<0.2 µM>	

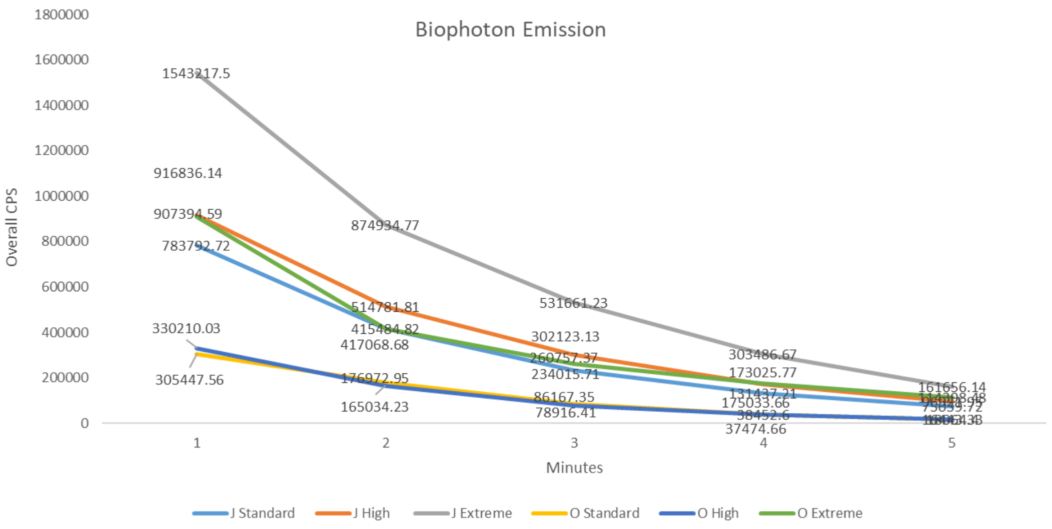

Figure S1. Overall photon count per secound of 'Oitol' and 'Joker' cucumber leaves treated with different levels of nitrate concentrations.

Table S2. Target genes, oligonucleotide primers and expected product sizes in RT-PCRs

Gene	Accession No	Forward primer (5'-3')	Reverse primer (5'-3')	size (bp)
MDHAR1	Csa3M775240*	CCGGTTCCACTGTCATAAAATTG	GATCATGGTAACATCAAGATCGTTG	212
MDHAR2	Csa6M451470*	TATCCAAGGAGGCGGTTG	CTCTTGGCAGGAAGATCTGC	202
MDHAR3	Csa3M099720*	TGGAGTGGCAGCAGGATATG	GAGGATCAATTCTATCCCTTTCTCTTC	235
MDHAR4	Csa5M524740*	TGAAGCACAGTGCATGCC	CCAACAACAACCATGTCAACA	192
APX1	CsaV3_6G047320	GCAGATCTGTACCAGCTTGCTG	CAGCAATTCCACAAAGTAGGAGTTATC	303
APX2	CsaV3_6G021870	CTTCGTCTCGCATGGCA	TGCCATTGAAGTTACTGGTGG	221
APX4	CsaV3_2G032090	CGACACGAGAACTAGTTGCACTAT	GAATGCTTGAGATGGATCAAAGAG	111
APX5	CsaV3_3G047650	AACTCGCAGCACAAAGTGGC	CGGACAGAACTGCCAGCTG	270
GR1	CsaV3_3G011090	TACGATCTCTGGCCGACAAGAG	CAGCACTGTTGGAATACACCCAT	182
GR2	CsaV3_7G027540	CGTGGAAAGATTCTGGATGAAC	GCTCTAGAATTTGCTGGCATC	216
GME2	Csa2M011460.1*	GTAGAAGGTGTCCTGAGATTG	TCTGAGTTACGACCACGG	173
GGP	Csa7M219200.1*	TTTCTTCTCCCACAGTGTTATG	TTCCTTCGTCCCAGTCATT	360
GalDH	Csa7M067450.1*	GTCTTGGCATCAACTTCTTC	TGCTCCTTGTCACCCTCT	177
GLDH	CsaV3_UNG143090*	GCCCTCCCAAATTCAAACC	GGGCAAGTAGTTTATCGCG	153
ACTIN	CsaV3_2G018090*	TCGTGCTTGACTCTGGTGATGG	ACAACCACTGCCGAACGGGAAA	171

*Primers are from Liu et al., 2019 [46], otherwise designed in this study.
